# Prenatal diagnosis of 9p distal deletion associated with subependymal cysts: A case report and literature review

**DOI:** 10.1097/MD.0000000000048420

**Published:** 2026-04-17

**Authors:** Yuanyuan Zhang, Fagui Yue, Tingting Qi, Ruizhi Liu

**Affiliations:** a Center for Reproductive Medicine and Center for Prenatal Diagnosis, First Hospital, Jilin University, Changchun, China; b Jilin Engineering Research Center for Reproductive Medicine and Genetics, Jilin University, Changchun, China.

**Keywords:** Chromosomal 9p deletion, chromosomal microarray analysis, pregnancy outcome, prenatal phenotype

## Abstract

**Rationale:**

Chromosome 9p deletions are frequently detected in postnatal cases presenting with diverse neurological symptoms. However, limited prenatal reports of these chromosomal disorders poses a challenge for genetic counseling during pregnancy.

**Patient concerns:**

A pregnant woman underwent amniocentesis for cytogenetic analysis and a chromosomal microarray analysis (CMA) because of imaging findings indicating subependymal cysts. The prenatal phenotypes were reviewed on the basis of previous reports.

**Diagnoses:**

The fetal karyotype was 46,XN,del(9)(p22). The CMA showed a 16.65-Mb deletion in the 9p24.3p22.2 region.

**Interventions:**

After genetic counseling, the couple chose to terminate the pregnancy.

**Outcomes:**

Fetal growth restriction and a single umbilical artery are common ultrasound findings detected in 9p deletions.

**Lessons:**

The prenatal genotype–phenotype of 9p deletion syndrome is complicated because of phenotypic diversity and incomplete penetrance. To offer better genetic counseling for such cases, ultrasonographic, cytogenetic, and molecular genetic results should be combined.

## 1. Introduction

Chromosomal deletions and duplications are associated with intellectual disability, developmental delay, autistic spectrum disorders, and congenital abnormalities.^[1]^ Chromosome 9p deletion syndrome (OMIM: 158170), which was first described by Alfi et al in 1973,^[2]^ is characterized by intellectual disability, developmental delay, trigonocephaly, upward-slanting palpebral fissures, midface hypoplasia, hypotonia, and occasionally gonadal dysgenesis in 46,XY individuals.^[3,4]^ The incidence of chromosome 9p deletion syndrome is estimated to be 1 in 50,000 newborns. Two thirds of these patients are female.^[5]^ Approximately half of 9p deletions are due to *de novo* deletions and the remaining half are due to unbalanced chromosomal rearrangements.^[6]^

Conventional cytogenetics can only detect chromosomal abnormalities at low resolution, causing difficulty in characterizing chromosome microscopic imbalances in detail, especially in cases of *de novo* unbalanced chromosomal rearrangements. However, molecular genetic techniques, such as chromosomal microarray analysis (CMA), can identify chromosomal copy number variants (CNVs) and breakpoints at the molecular level more clearly, providing deeper insight into genotype–phenotype correlations in the clinic. Therefore, the diagnostic yield of CMA is becoming indispensable for delineating chromosomal deletions and duplications, especially in prenatal setting.

Currently, the postnatal phenotypes associated with 9p deletions are well documented. However, the association between prenatal phenotypes and 9p deletions remains poorly defined because of limited reports, complicating prenatal genetic counseling for these pregnancies in clinical practice. In this study, we present one case of prenatal diagnostic *de novo* distal 9p deletion associated with ultrasound anomalies. We also conducted a literature review of prenatal cases involving 9p deletions and investigated the genotype–phenotype correlations.

## 2. Case presentation

### 2.1. Clinical information

**Figure 1.**
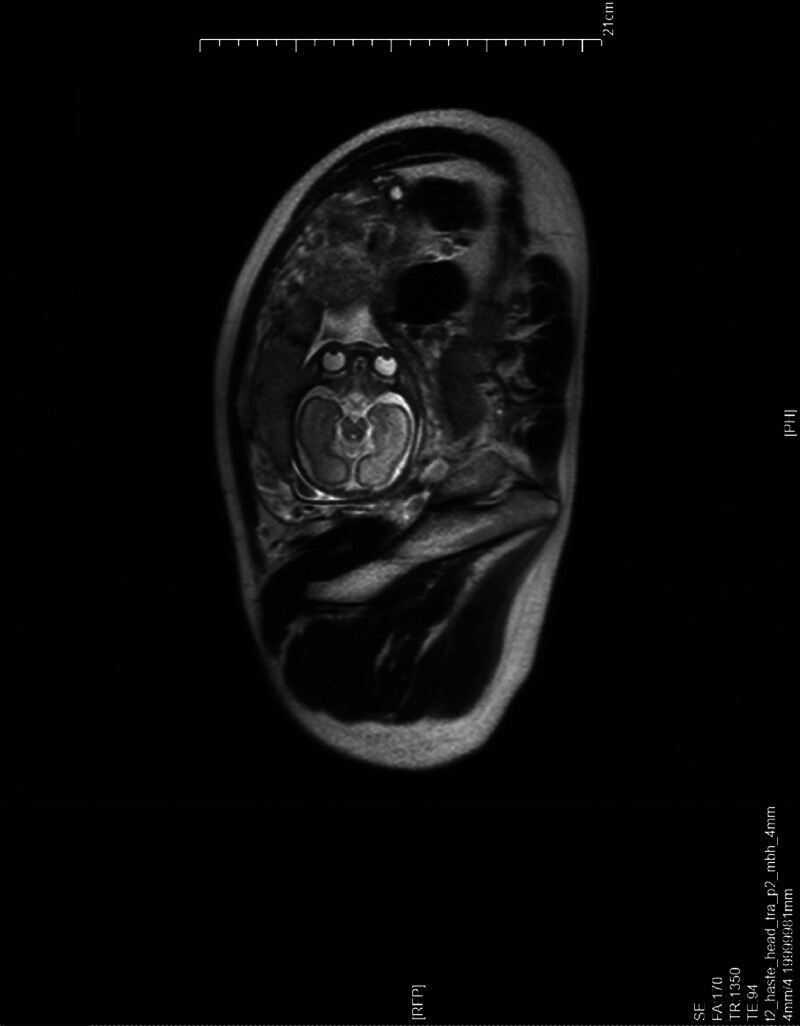
Fetal MRI at 27 weeks of gestation shows the subependymal cysts in the fetus. MRI, magnetic resonance imaging.

**Figure 2.**
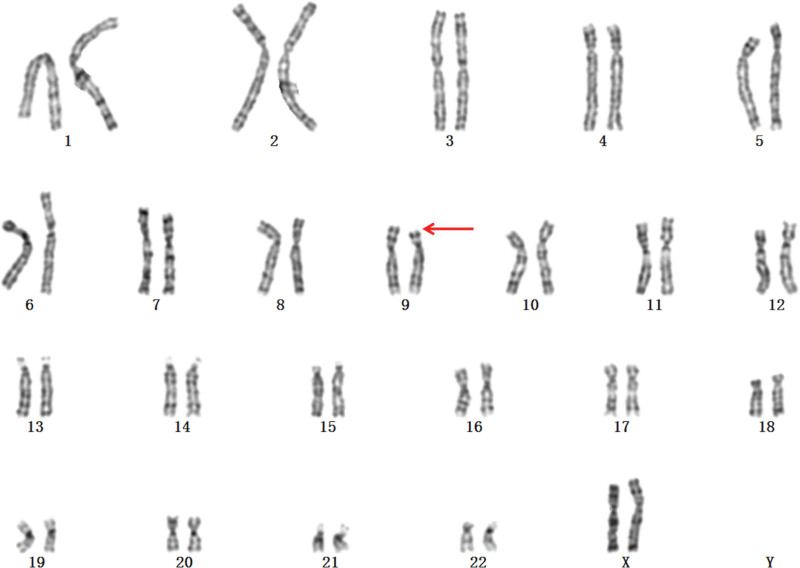
The karyotype of the fetus was 46,XN,del(9)(p22).

**Figure 3.**
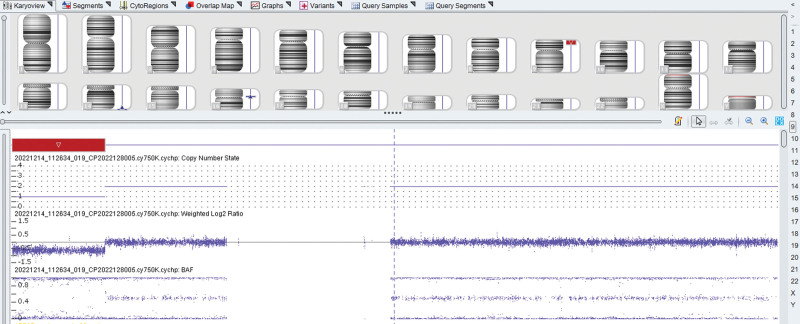
CMA revealed a 16.65-Mb deletion in the region of 9p24.3p22.2.

A 30-year-old pregnant woman (gravida 1, para 0) at 27 weeks of gestation was referred to our hospital on December 4, 2022 because of abnormal prenatal ultrasound findings indicating fetal subependymal cysts at a local hospital. To further evaluate the fetal ultrasound anomaly, fetal magnetic resonance imaging (MRI) was performed at our center, and it confirmed the ultrasound findings (Fig. 1). After signing an informed consent form, the patient underwent amniocentesis at 27 weeks of gestation at our center for cytogenetic analysis and CMA. G-banding karyotype analysis of amniotic fluid showed 46, XN,del(9)(p22) (Fig. 2). CMA using the CytoScan 750K array (Affymetrix, Santa Clara, CA, USA) identified arr[GRCh38]9p24.3p22.2 (208,455–16,855,040) × 1, which indicated a 16.65-Mb deletion in the 9p24.3p22.2 region (Fig. 3). The karyotypes of the parents were normal, which suggested a *de novo* 9p deletion in the fetus. Following genetic counseling, the couple chose to terminate the pregnancy. The pregnant woman denied any exposure to alcohol, teratogenic agents, irradiation, or infectious diseases during this pregnancy. Our study protocol was approved by the Ethics Committee of the First Hospital of Jilin University (2021–706), and written informed consent for publication was obtained from the couple.

### 2.2. Cytogenetic analysis

The pregnant woman underwent amniocentesis, and a total of 30 mL of amniotic fluid cells were collected. A routine cytogenetic analysis was performed on G-band metaphases at 400 to 500 banding resolution, and this required 20 mL of cultured amniotic fluid cells following the standard protocols in our laboratory. In accordance with the International System for Human Cytogenetic Nomenclature 2016, 20 metaphases retrieved from all samples were analyzed.

### 2.3. CMA

A volume of 10 mL of uncultured amniotic fluid cells was collected for the extraction of genomic DNA using the QIAamp® DNA Blood Mini Kit (Qiagen, Inc., Hilden, Germany) by following the manufacturer’s protocol. The procedure was conducted using the CytoScan 750K array (Affymetrix, Santa Clara, CA, USA) by following the manufacturer’s protocol and as described in our previous study.^[7]^ This process included genomic DNA extraction, digestion and ligation, polymerase chain reaction amplification, polymerase chain reaction product purification, quantification and fragmentation, labeling, array hybridization, washing, and scanning. Thresholds for genomewide screening were set at ≥ 100 kb for gains and losses. All detected CNVs were comprehensively evaluated by comparing them with published literature and public databases, including the Database of Genomic Variants (DGV, http://dgv.tcag.ca/dgv/app/home), Database of Chromosomal Imbalance and Phenotype in Humans using Ensemble Resources (DECIPHER, https://www.deciphergenomics.org/), Clinical Genome Resource (ClinGen, http://www.clinicalgenome.org/), ClinVar (https://www.ncbi.nlm.nih.gov/clinvar/), PubMed (https://pubmed.ncbi.nlm.nih.gov/), and OMIM (https://omim.org/). The CNVs were classified as pathogenic, likely pathogenic, variants of unknown significance, likely benign, and benign. Genomic positions refer to the Human Genome assembly Dec. 2013 (GRCh38/hg38).

## 3. Discussion

We report a rare prenatal case with a *de novo* 16.65-Mb deletion in the 9p24.3p22.2 region. Subependymal cysts were identified in the fetus by a fetal MRI examination. After prenatal genetic counseling, the couple chose to terminate the pregnancy. To the best of our knowledge, this is the first reported case of prenatally detected 9p deletion with subependymal cysts.

Since chromosome 9p deletion syndrome was initially described in the 1970s, more than 100 cases with detailed phenotypes have been reported. Patients with 9p deletion show varied clinical manifestations, ranging from mild developmental delays to severe malformations. The most frequent phenotypes in all 9p deletion carriers are developmental delay, speech delay, and motor delay. Additional shared phenotypes with high frequency include trigonocephaly, midface hypoplasia, short palpebral fissures, low-set ears, short/flat nose, anteverted nostrils, thin upper lip, long philtrum, short/broad neck, and broad internipple distance.^[8]^ The cardiac phenotypes are highly heterogeneous among 9p deletion carriers, ranging from subtle to severe.^[9]^ Reversed sex or impaired gonadal development has also been observed in some patients. The incidence rate of ambiguous genitalia was estimated to be 70% in individuals with 9p deletion.^[10,11]^ Sex reversal in some individuals with 9p deletions might also lead to a sex bias, with considerable enrichment in female.^[8]^ In general, as one of the CNVs associated with broad phenotypic features and frequently detected postnatally, the 9p deletion is characterized by phenotypic diversity and incomplete penetrance.

The clinical manifestations involving 9p deletion are widely believed to be affected by specific chromosomal loci and the deleted size, which makes the genotype–phenotype correlation complicated. In recent years, an increasing number of studies have focused on investigating the association between phenotype data and the critical regions of 9p deletions in depth. Sams et al collected genetic data from 719 individuals in the worldwide 9p Network Cohort and found that most breakpoints occurred in bands 9p22 and 9p24, which accounted for 35% and 38% of all breakpoints, respectively. Bands 9p11 and 9p12 had the fewest breakpoints, each accounting for 0.6%.^[8]^ The critical region for 9p deletion syndrome is thought to map to a 4 to 6 Mb region in 9p22–p23, which is commonly responsible for the clinical phenotypes, especially trigonocephaly.^[6,12]^ Hauge et al and Hou et al showed that deletions in the 9p23 region played a critical role in abnormal phenotypes in 9p deletion.^[4,13]^ A study by Mohamed et al enhanced the fact and they found that a minimal 11.8-kb region in 9p23 was the most critical region for trigonocephaly in 9p deletion syndrome.^[10]^ Meanwhile, the distal 9p24.3 deletion is considered to have a close association with intellectual disability, delayed speech, behavioral problems, 46,XY reversed sex (female external genitalia), and ambiguous genitalia in both sexes.^[3,4]^ It was proposed that the critical region extends to 9p24.3 when sex reversal and gonadal dysgenesis are included in the 9p deletion.^[11,14]^ In addition, a comparison of 9p deletions in the literature showed that 9p24 deletion might be associated with autism spectrum disorder or behavioral problems.^[3,15–17]^ Notably, the deletion of distal regulatory elements can perturb proximal genes, thereby contributing to the atypical phenotypes associated with small 9p terminal deletions.^[14]^ As further research is performed, the genotype–phenotype correlation of 9p deletion will be clarified more precisely.

**Figure 4.**
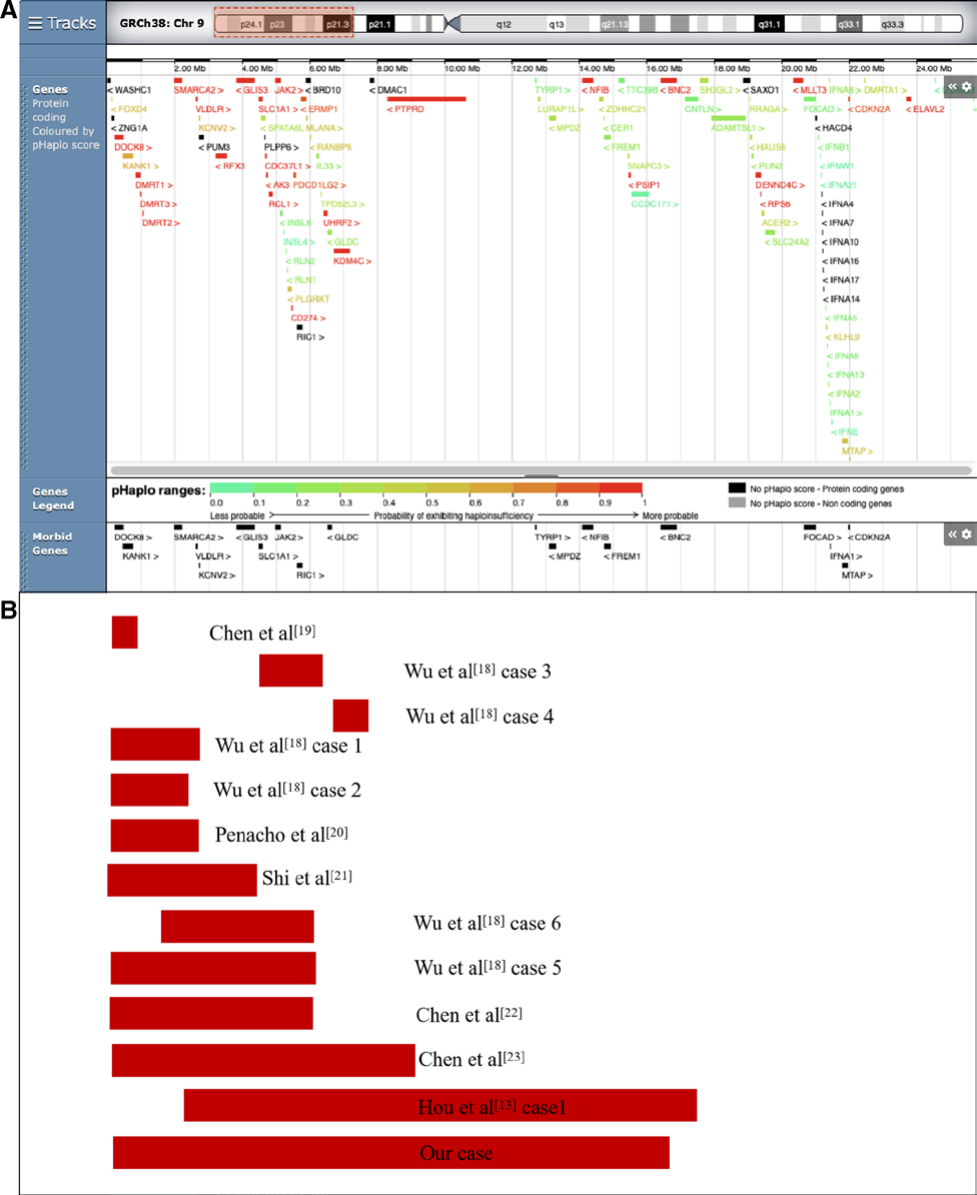
Scale representation of the deleted region in the short arm of chromosome 9p24.3p22.2 (https://www.deciphergenomics.org/): (A) Genes involved in the 9p24.3p22.2 region; (B) previously described 9p deletions in the prenatal period and deleted fragments in our case. Genomic parameters are from GRCh38/hg38.

To date, most reported cases of 9p deletion have been identified postnatally, and prenatal cases are particularly uncommon. To improve our understanding of the prenatal phenotypes associated with 9p deletion, we performed a comprehensive literature review and summarized the clinical manifestations of prenatal cases with similar 9p deletions in the literature (Table 1, Fig. 4).^[13,18–27]^ The gestational age of these case was between 12 and 25 weeks. The deletion sizes varied from 0.15 to 16.65-Mb. Of the 18 deletion cases, 13 were *de novo*, 3 were of unknown inheritance, and 2 were parentally inherited. The indications for prenatal diagnosis included abnormal ultrasound findings (11/18), a risk of Down syndrome (7/18), and advanced maternal age (2/18). The types and frequencies of abnormal prenatal phenotypes in the literature and our study were as follows: fetal growth restriction (3/18), single umbilical artery (3/18), bilateral ventriculomegaly (2/18), omphalocele (2/18), genitourinary anomalies (2/18), polyhydramnios (1/18), left heart hypoplasia (1/18), craniofacial abnormalities (1/18), increased nuchal translucency (1/18), and subependymal cysts (1/18). Based on these findings, fetal growth restriction and a single umbilical artery were the most common ultrasound findings detected in 9p deletions.

**Table 1:** The prenatal phenotypes of present cases and published literature with 9p deletion.

Case No.	Age	Gravida para	Gestational age (w)	Indications for prenatal diagnosis	Ultrasound findings during pregnancy	Region	Karyotype	CMA results (GRCh38)	Size (Mb)	Inheritance	Pregnancy outcome		References
											Gestational age	Birth weight (g)	
1	32	Unknown	20+	Down syndrome risk of 1/150	No evident anomalies	9p24.2p24.3	Unknown	9p24.3p24..2 (204193–2766782) × 1	2.56	*de novo*	TOP		Wu et al^[18]^ case 1
2	30	Unknown	19+	Down syndrome risk of 1/500	No evident anomalies	9p24.2p24.3	Unknown	9p24.3p24..2 (204193–2430956) × 1	2.23	*de novo*	TOP		Wu et al^[18]^ case 2
3	29	Unknown	20+	Down syndrome risk of 1/90	No evident anomalies	9p24.1p24.2	46,XY	9p24.2p24..1 (4483336–6359510) × 1	1.88	pat	Live birth		Wu et al^[18]^ case 3
4	34	Unknown	22+	Down syndrome risk of 1/100	No evident anomalies	9p24.1	46,XY	9p24..1 (6714941–7733826) × 1	1.02	*de novo*	Live birth		Wu et al^[18]^ case 4
5	32	Unknown	22+	Down syndrome risk of 1/580	No evident anomalies	9p24.1p24.3	Unknown	9p24.3p24..1 (271257–6272575) × 1	6.00	*de novo*	TOP		Wu et al^[18]^ case 5
6	31	Unknown	24+	Down syndrome risk of 1/430	No evident anomalies	9p24.1p24.3	Unknown	9p24.3p24..1 (1579408–6055556) × 1	4.48	*de novo*	TOP		Wu et al^[18]^ case 6
7	35	G1P0	16+	AMA, ultrasound abnormalities	Ventriculomegaly	9p24.3 9p22.1p24.3	46,XY,inv dup del(9) (:p22.1 → p24.3: p24.3 → qter)	9p24.3p24..3 (271057–974003) × 1 9p24.3p22..1 (1036210–19396810) × 3	0.70 18.36	mat	TOP		Chen et al^[19]^
8	31	Unknown	18+	Ultrasound abnormalities	FGR	9p24.2pter 9q34.3qter 9p24.1	46,XX,r(9)(p24q34)	9pterp24..2 (163131–2729722) × 1 9q34.3qter(135631456–138231605) × 1 9p24..1 (5090443–5235765) × 1	2.57 2.60 0.15	*de novo*	TOP		Penacho et al^[20]^
9	32	G4P1	22+	Ultrasound abnormalities	Mild dilation of bilateral lateral ventricles, polyhydramnios	9p24.2p24.3	46,XX,der(9)dir dup(9)(p21.3p24.2), del(9)(p24.2p24.3)	9p24.2p24..3 (10001–4442364) × 1 9p21.3p24..2 (4454279–25126277) × 3	4.43 20.67	*de novo*	TOP		Shi et al^[21]^
10	32	G2P0	25+	Down syndrome risk: 1/147, ultrasound abnormalities	FGR	9p24.1p24.3	46,XX	9p24.3p24..1 (198350–6256729) × 1	6.06	*de novo*	TOP		Chen et al^[22]^
11	34	Unknown	17+	AMA	No evident anomalies	9p23p24.3	46,XY, del(9)(p23)[8]/46,XY[17]	9p24.3p23(204193–9114563) × 1.55	8.91	*de novo*	41	3020	Chen et al^[23]^
12	31	G3P1	17+	Ultrasound abnormalities	Omphalocele	9p13	46,XY	Unknown	Unknown	*de novo*	TOP		Hou et al^[24]^
13	30	G2P1	17+	Ultrasound abnormalities	Omphalocele	9p22.2p24.2	46,XX	9p24.2p22..2 (2267812–17466907) × 1	15.19	*de novo*	TOP		Hou et al^[13]^ case1
14	Unknown	Unknown	24+	Ultrasound abnormalities	Ambiguous genitalia with clitoral hypertrophy	9p22pter	46,XY	Unknown	Unknown	Unknown	TOP		Vialard et al^[25]^ case1
15	47	Unknown	24+	Ultrasound abnormalities	Left heart hypoplasia, single umbilical artery	9p22pter	46,XX	Unknown	Unknown	Unknown	TOP		Vialard et al^[25]^ case2
16	Unknown	Unknown	12+	Ultrasound abnormalities	Micrognathia, an enlarged posterior fossa, bilateral pes equinovarus, single umbilical artery, a thickened umbilical cord, sex reversal	9p24	46,XY,der(9)t(3;9)(p14.2;p24)	Unknown	Unknown	Unknown	TOP		Witters et al^[26]^
17	29	G1P0	22+	Ultrasound abnormalities	Increased NT, single umbilical artery, partial agenesis of the cerebellar vermis, bilateral cystic choroid plexus, facial dysmorphisms, FGR	9p24.3	46,XX,der(9)t(9;17)(p24.3;q24.3)	Unknown	2.4	*de novo*	TOP		Brisset et al^[27]^
18	30	G1P0	17+	Ultrasound abnormalities	Subependymal cysts	9p22.2p24.3	46,XN	9p24.3p22..2 (208455–16855040) × 1	16.65	*de novo*	TOP		Our case

Genomic parameters are from GRCh38/hg38.

AMA = advanced maternal age, CMA = chromosomal microarray analysis, FGR = fetal growth restriction, mat = maternal inherited, NT = nuchal translucency, TOP = termination of pregnancy, pat = paternal inherited; w = weeks.

**Figure 5.**
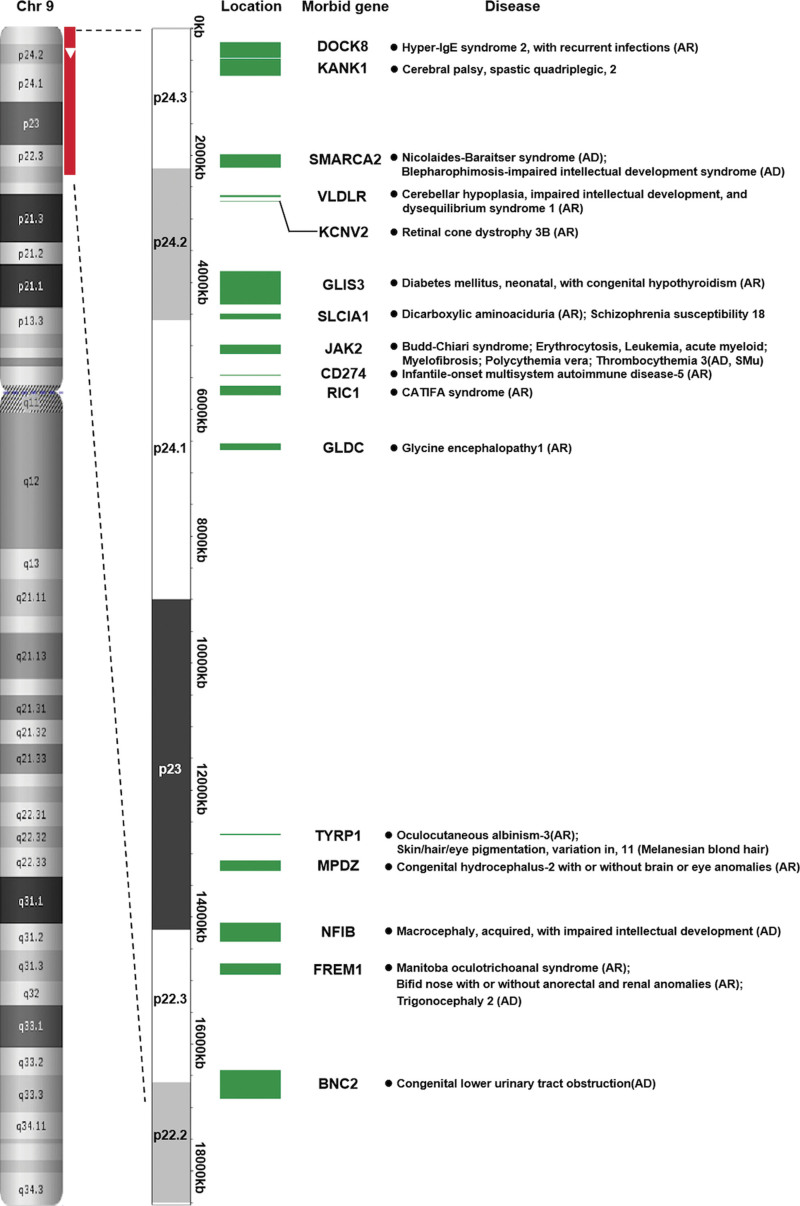
Schematic maps of morbid genes in the region of 9p22.2–9p24.3 and the associated diseases. AR = autosomal recessive; AD = autosomal dominant; SMu = somatic mutation.

According to the DECIPHER database, 49 protein coding genes were located in the 9p24.3p22.2 deletion in our case, 16 of which are morbid genes associated with clinical diseases (Fig. 5). Our case presented with subependymal cysts, which is a minor central nervous system anomaly. Combined with this prenatal phenotype, we determined the potentially pathogenic genes on the basis of their functions and implications in different processes, especially in neurofunctional development. *VLDLR* (OMIM: 192977) is a receptor for reelin and plays a crucial role in brain development. Mutations in *VLDLR* can lead to cerebellar hypoplasia, intellectual disability, and disequilibrium syndrome.^[28]^ *NFIB* (OMIM: 600728) is essential for lung maturation and brain development. According to ClinGen database records, there is sufficient evidence for haploinsufficiency (HI score: 3) in this gene. Deletion of this gene can cause syndromic neurodevelopmental disorder, which is associated with the central nervous system, including intellectual disability, autism, and other features, such as dysmorphisms and/or congenital malformations. Schanze et al.^[29]^ also assumed that *NFIB* haploinsufficiency can cause intellectual disability and macrocephaly. *DOCK8* (OMIM: 611432) is a member of the DOCK180-related protein family and expressed in several human tissues, including the adult and fetal brain.^[30]^ Deletions of *DOCK8* are associated with mental retardation and developmental disabilities in individuals with 9p CNVs. Variants in the *KANK1* gene are associated with cerebral palsy, autism, nephrotic syndrome, and male congenital genitourinary anomalies.^[31]^ *RIC1* mutations can cause CATIFA syndrome, which is a rare neurodevelopmental disorder characterized by cleft lip, cataract, tooth abnormality, intellectual disability, facial dysmorphism, and attention deficit hyperactivity disorder.^[32]^ *SMARCA2* mutations cause blepharophimosis-impaired intellectual development syndrome and Nicolaides–Baraitser syndrome, and some of their features overlap with 9p deletion syndrome. Therefore, *SMARCA2* mutations are likely a contributor to this syndrome.^[31]^ Additionally, *GLDC* (OMIM: 238300) is associated with glycine encephalopathy. *DMRT1* (OMIM: 602424) and *DMRT3* (OMIM: 614754) are implicated in disorders of sex development, and 9p deletion causes sex reversal in XY fetuses but not in XX fetuses.^[20]^ *FREM1* (OMIM: 608944) is probably associated with metopic craniosynostosis and trigonocephaly.^[12]^ A recent study showed that haploinsufficiency of *DOCK8*, *KANK1,* and *VLDLR* may be responsible for neurological manifestation in individuals with 9p deletion syndrome.^[33]^ Six genes (*CDC37L1, NFIB, PTPRD, RFX3, SMARCA2,* and *UHRF2*) with a probability of loss-of-function intolerance (pLI) > 0.9 were enriched for deletions in individuals with neurodevelopmental disorders, which indicated that the dosage of these genes may play a role in these disorders.^[8]^ These genes play crucial roles in the clinical phenotypes observed in 9p deletions, especially in neurofunctional defects. The ultrasound anomaly in our study was probably due to the 9p24.3p22.2 deletion and haploinsufficiency of specific genes, while further investigation is still required to clarify the correlation between prenatal phenotype and 9p deletion.

Traditional cytogenetic techniques play critical roles in identifying chromosomal balanced structural rearrangements that cannot be detected by CMA. However, CMA allows the detection of submicroscopic imbalances in the kilobase range because of high resolution. The combined application of these 2 techniques has complementary advantages, which could offer more genetic testing details. In this study, conventional karyotyping identified 9p deletion while CMA further localized and precisely defined the deleted content, which is beneficial for prenatal genetic counseling and clinical management. In our study, the fetus carried a *de novo* chromosomal 9p deletion. Although *de novo* deletions have a low risk of recurrence, genetic counseling and prenatal diagnosis are still necessary if the couple intend to conceive again.

## 4. Conclusion

In this study, we report the clinical data and molecular findings of a prenatal case with a *de novo* distal 9p deletion accompanied by subependymal cysts. Fetal growth restriction and a single umbilical artery are common recurrent ultrasound findings associated with 9p deletions, which would provide more information for prenatal genetic counseling. The combined application of karyotyping and CMA plays a critical role in detecting this chromosomal abnormalities during prenatal diagnosis, thereby facilitating the clinical management of high-risk pregnancies. Given that 9p deletion is characterized by phenotypic diversity and incomplete penetrance, additional studies are needed to further elucidate the prenatal genotype-phenotype correlation.

## Acknowledgments

We thank Ellen Knapp, PhD, from Edanz (https://jp.edanz.com/ac) for editing a draft of this manuscript.

## Author contributions

**Conceptualization:** Yuanyuan Zhang, Ruizhi Liu.

**Writing – original draft:** Yuanyuan Zhang.

**Formal analysis:** Fagui Yue.

**Methodology:** Fagui Yue.

**Data curation:** Tingting Qi.

**Supervision:** Ruizhi Liu.

**Writing – review & editing:** Ruizhi Liu.
